# Intravenous Thrombolysis by Telestroke in the 3- to 4.5-h Time Window

**DOI:** 10.3389/fneur.2021.756062

**Published:** 2021-11-26

**Authors:** Erik Simon, Matin Forghani, Andrij Abramyuk, Simon Winzer, Claudia Wojciechowski, Lars-Peder Pallesen, Timo Siepmann, Heinz Reichmann, Volker Puetz, Kristian Barlinn, Jessica Barlinn

**Affiliations:** ^1^Department of Neurology, University Hospital Carl Gustav Carus, Technische Universität Dresden, Dresden, Germany; ^2^Department of Radiology, Institute of Neuroradiology, Carl-Thiem-Klinikum Cottbus, Cottbus, Germany

**Keywords:** telemedicine, thrombolysis, stroke, acute stroke therapy, stroke network

## Abstract

**Background:** While intravenous thrombolysis (IVT) in ischemic stroke can be safely applied in telestroke networks within 3 h from symptom onset, there is a lack of evidence for safety in the expanded 3- to 4. 5-h time window. We assessed the safety and short-term efficacy of IVT in acute ischemic stroke (AIS) in the expanded time window delivered through a hub-and-spoke telestroke network.

**Methods:** Observational study of patients with AIS who received IVT at the Stroke Eastern Saxony Telemedical Network between 01/2014 and 12/2015. We compared safety data including symptomatic intracerebral hemorrhage (sICH; according to European Cooperative Acute Stroke Study II definition) and any intracerebral hemorrhage (ICH) between patients admitted to telestroke spoke sites and patients directly admitted to a tertiary stroke center representing the hub of the network. We also assessed short-term efficacy data including favorable functional outcome (i.e., modified Rankin Scale ≤ 2) and National Institutes of Health Stroke Scale (NIHSS) at discharge, hospital discharge disposition, and in-hospital mortality.

**Results:** In total, 152 patients with AIS were treated with IVT in the expanded time window [spoke sites, *n* = 104 (26.9%); hub site, *n* = 48 (25.9%)]. Patients treated at spoke sites had less frequently a large vessel occlusion [8/104 (7.7) vs. 20/48 (41.7%); *p* < 0.0001], a determined stroke etiology (*p* < 0.0001) and had slightly shorter onset-to-treatment times [210 (45) vs. 228 (58) min; *p* = 0.02] than patients who presented to the hub site. Both cohorts did not display any further differences in demographics, vascular risk factors, median baseline NIHSS scores, or median baseline Alberta stroke program early CT score (*p* > 0.05). There was no difference in the frequency of sICH (4.9 vs. 6.3%; *p* = 0.71) or any ICH (8.7 vs. 16.7%; *p* = 0.15). Neither there was a difference regarding favorable functional outcome (44.1 vs. 39.6%; *p* = 0.6) nor median NIHSS [3 (5.5) vs. 2.5 (5.75); *p* = 0.92] at discharge, hospital discharge disposition (*p* = 0.28), or in-hospital mortality (9.6 vs. 8.3%; *p* = 1.0). Multivariable modeling did not reveal an association between telestroke and sICH or favorable functional outcome (*p* > 0.05).

**Conclusions:** Delivery of IVT in the expanded 3- to 4.5-h time window through a telestroke network appears to be safe with equivalent short-term functional outcomes for spoke-and-hub center admissions.

## Introduction

Although the implementation of endovascular therapy (EVT) in the treatment of acute ischemic stroke (AIS) has a largely improved prognosis of the disease, intravenous thrombolysis (IVT) using tissue plasminogen activator continues to be the mainstay of acute care of patients with AIS and remains of great importance for prevention of long-term disability (1, 2). The efficacy and safety of IVT are primarily time-dependent and the benefit increases the earlier and faster the therapy is initiated (3).

The widespread availability of evidence-based stroke therapies, regardless of geographical barriers, is still a challenge of acute stroke care (4). It has been shown that telemedicine can overcome this challenge and improve the care of patients with stroke through the identification of patients in need of IVT or EVT and further rescue therapies (5–7). This is reflected by the fact that telestroke networks meanwhile achieve similar rates of IVT and transfers for EVT compared with neurological stroke centers (8).

We have recently shown that IVT delivered through telestroke network is not inferior in terms of safety and efficacy to tissue-type plasminogen activator (tPA) provided at specialized stroke centers for the treatment of AIS in the 3-h time window (9). However, while recent data even suggest a benefit of IVT up to 9 h from symptom onset using advanced imaging techniques that are commonly reserved to dedicated stroke centers, there is still a lack of evidence regarding its safety and efficacy in the regularly approved 3- to 4.5-h therapeutic time window in the telestroke setting (9–12). In view of these considerations, we aimed to investigate the safety and short-term efficacy of IVT in the 3- to 4.5-h time window for treatment of AIS in a telestroke network.

## Methods

### Study Design and Telestroke Network

**Figure 1:**
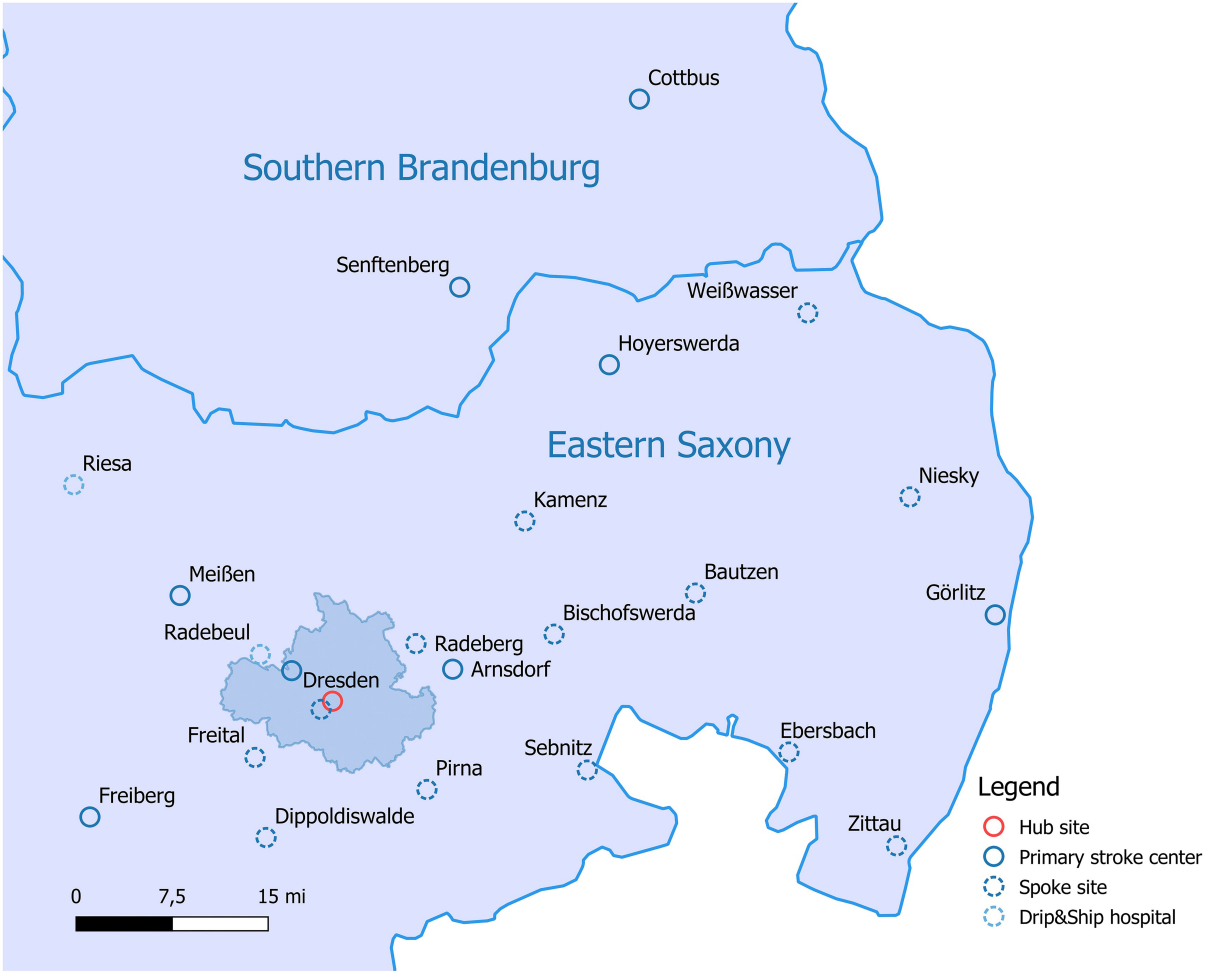
Map of the Stroke East Saxony Telemedical Network in the eastern part of Saxony, Germany.

We performed an observational study using prospectively collected data from a large hub-and-spoke telestroke network in Saxony, Germany. The Stroke Eastern Saxony Telemedical Network (SOS-TeleNET) founded in 2007 comprises 13 spoke sites and provides telestroke care to ~1.000 patients per year (Figure 1). The Department of Neurology of the University Hospital Carl Gustav Carus in Dresden serves as the main hub for each of the spokes. At the time of the study period (01/2014–12/2015), the SOS-TeleNET also included two secondary care hospitals, which served as additional Neurology hub sites. One site performed teleconsultations 3 days per month, the other site provided neurosurgical care of patients with stroke, but no teleconsultation service. The distance between the main hub and the surrounding spoke sites is between 15 km (about 10 mi) and up to 120 km (about 75 mi).

The video-based evaluation of the neurological status and immediate review of the cerebral imaging transmitted *via* virtual private network was performed 24/7 by a stroke neurologist at the main hub site using either a stationary telemedical unit (VIMED® DOC, MEYTEC GmbH Medizinsysteme, Werneuchen) or wireless Universal Mobile Telecommunications System (VIMED® UMTS 2, MEYTEC GmbH Medizinsysteme, Werneuchen) outside working hours. Neuroimages were transmitted in DICOM (Digital Imaging and Communications in Medicine) format and temporarily stored on a certified PACS (Picture Archiving and Communication System) server. Imaging findings and stroke cases potentially amenable to interventional therapies were discussed with a neuroradiologist who was available 24/7. At spoke sites, a mobile telemedical system (VIMED® TELEDOC, MEYTEC GmbH Medizinsysteme, Werneuchen) located in the emergency room was used for teleconsultations. Indications for telestroke consultations comprised suspected stroke within a therapeutic time window up to 24 h from symptom onset, intracranial hemorrhage, brainstem symptoms, unclear qualitative or quantitative disturbances of consciousness, unclear clinical or diagnostic status, and progressive stroke.

All spoke sites followed standard operating procedures provided by the SOS-TeleNET and were guided by current stroke guidelines (13, 14). Also, annual quality assurance audits were conducted at all spoke sites to ensure evidence-based and high-quality stroke care.

As the standard of care, serial National Institutes of Health Stroke Scale (NIHSS) scores and Alberta stroke program early CT score (ASPECTS) on baseline CT scan were obtained in all patients. Guided by clinical and imaging findings, stroke neurologists ultimately gave recommendations regarding treatment with IVT and transfer to the hub site for further treatment evaluation. Patient data, namely, demographical information, medical history, stroke-related information, and treatment specifics and characterization of stroke etiology were retrieved from prospective teleconsult summaries and the institutional stroke care quality registry. Additional information was extracted retrospectively from all available sources, namely, the hospitals' electronic patient databases and admission, follow-up, and discharge summaries.

### Patient Outcomes

To evaluate the safety and short-term efficacy of IVT with tPA in patients with telestroke, we compared data from patients presented to the spoke sites with that of patients primarily presented to the hub site. Safety outcomes included symptomatic intracerebral hemorrhage (sICH), defined as any intracerebral hemorrhage (ICH) on 12- to 36-h follow-up CT scan that was causatively associated with a four-point worsening of NIHSS, and any ICH according to the radiographic hemorrhagic transformation classification (15). For this purpose, all imaging data were prospectively reviewed by a board-certified neuroradiologist (A.A.) who was blinded to group allocation and clinical information. We also assessed favorable (i.e., modified Rankin Scale, mRS ≤ 2) functional outcome at discharge, NIHSS at discharge, in-hospital mortality, and hospital discharge disposition.

### Statistical Analysis

Continuous variables are presented as mean ± SD and non-continuous variables as median (interquartile range, IQR) or percentage. Between-group comparisons were conducted with the use of *t*-test, Mann-Whitney-*U*-test, chi-squared-test, and Fisher's exact-test,where appropriate.

A multivariable logistic regression analysis with a stepwise forward selection procedure was conducted to explore the association between telestroke and sICH or favorable functional outcome. Candidate variables were *a priori* selected according to their known predictive association with ICH (i.e., age, history of atrial fibrillation, concomitant therapeutic anticoagulation or antiplatelet therapy, onset-to-treatment time, admission glucose, baseline systolic blood pressure, baseline ASPECTS, and baseline NIHSS) or functional outcome (i.e., age, baseline NIHSS, baseline ASPECTS, onset-to-treatment time, and large vessel occlusion), and entered in the final model at *p* < 0.2. We also performed a sensitivity analysis, considering only variables that emerged significantly different in the between-group comparisons.

Available case analysis was used for any missing data on baseline parameters. *p*-value was considered significant at <0.05. Adjusted odds ratios (ORs) are presented with corresponding 95% CI. All analyses were computed with SPSS (Statistical Package for Social Sciences, version 20.0, IBM, Armonk, New York).

## Results

### Study Population

**Figure 2:**
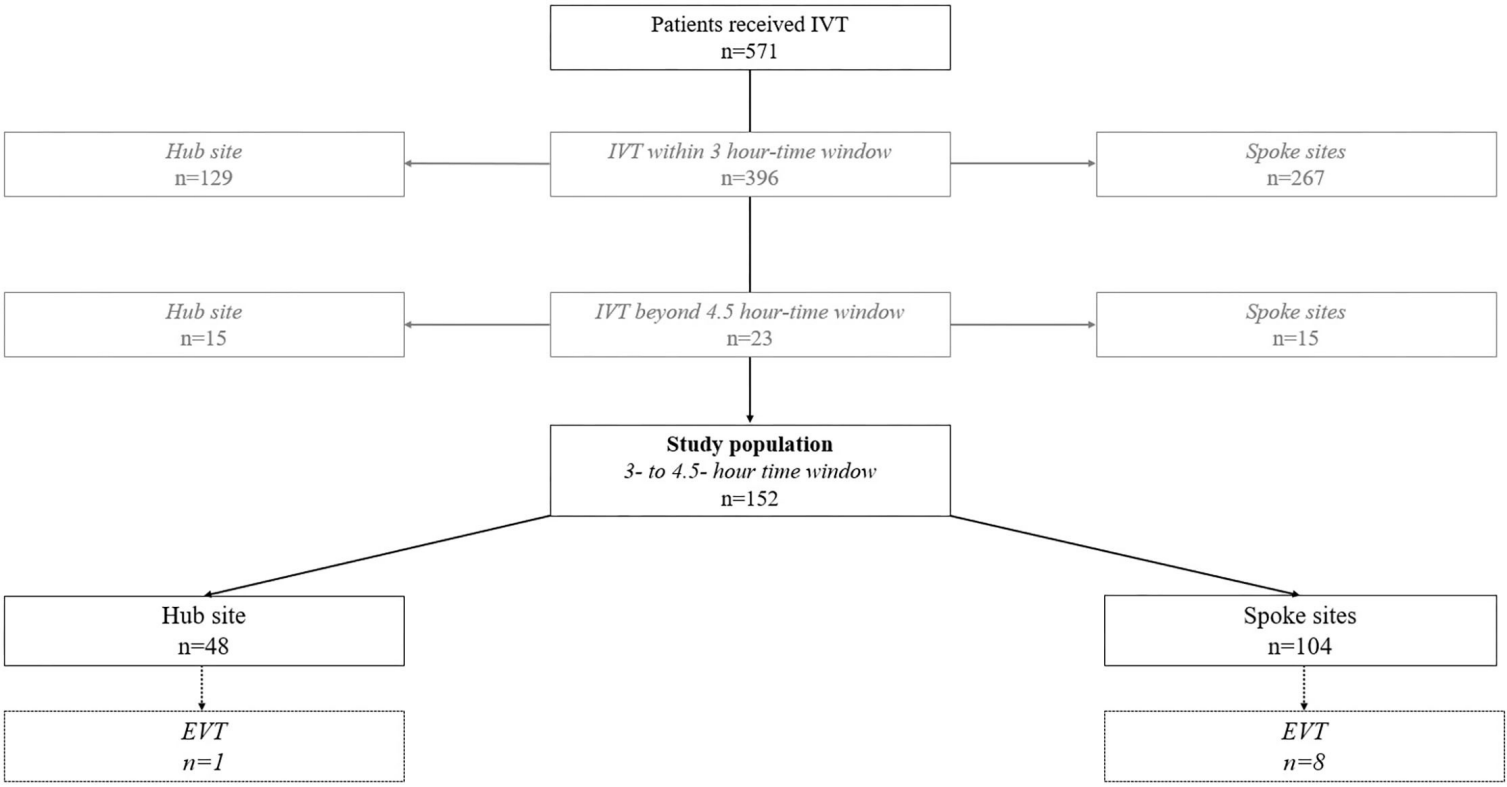
Flow chart of the study population. IVT, intravenous thrombolysis; EVT, endovascular therapy.

During the 2-year study period, a total of 571 patients with ischemic stroke received IVT within the SOS-TeleNET (spoke sites, *n* = 386; hub site, *n* = 185). Of these patients, 396 were treated within the 3-h time window [spoke sites, *n* = 267 (69.2%); hub site, *n* = 129 (69.7%)] and 23 beyond the 4.5-h time window [spoke sites, *n* = 15 (3.9%); hub site, *n* = 8 (4.3%)]. The final study population consisted of 152 patients with AIS who were treated in the 3- to 4.5-h time window [spoke sites, *n* = 104 (26.9%); hub site, *n* = 48 (25.9%)]. Eight of 104 (7.7%) patients with telestroke were subsequently transferred for potential EVT or advanced stroke care to the hub site (two eventually underwent EVT). In the stroke center cohort, 1/48 (2.1%) patients underwent EVT (Figure 2).

The mean age of the study population was 74 ± 12.3 years, 50% were men, baseline NIHSS scores was 7 (IQR, 8) points and baseline ASPECTS 10 (IQR, 1) points. Patients treated at spoke sites less frequently exhibited a large vessel occlusion [8/104 (7.7) vs. 20/48 (41.7%); *p* < 0.0001] and well-defined stroke etiology (*p* < 0.0001), and had slightly shorter onset-to-treatment times [210 (45) vs. 228 (58) min; *p* = 0.02] than patients who presented to the hub site. Further baseline characteristics, namely, demographics, vascular risk factors, and clinical and imaging parameters were well-balanced among both groups. Table 1 illustrates the corresponding baseline data of the study population.

**Table 1:** Clinical characteristics, process times, and stroke etiologies of the study population.

	**Telestroke (*n* = 104)**	**Stroke center (*n* = 48)**	***p-*value**
**Demographics**			
Gender, male, *n* (%)	52 (50)	24 (50)	1.0
Age, years, mean ±σ	73.3 ± 12.7	75.5 ± 11.4	0.32
**Initial stroke severity, median (IQR)**			
NIHSS	8 (9)	6.5 (6.8)	0.28
ASPECTS	10 (1)	10 (1)	0.99
**Clinical baseline values**, $\bar{\text{x}}$ **±σ**			
Serum glucose, mmol/L	7.1 ± 2.7	7.1 ± 2.4	0.85
Initial systolic blood pressure	167 ± 31.3	170 ± 34	0.55
Initial diastolic blood pressure	87 ± 27	89 ± 21.5	0.36
Pre-IVT systolic blood pressure	160 ± 29	156 ± 26	0.24
Pre-IVT diastolic blood pressure	83 ± 19	81 ± 15.8	0.55
**Vascular risk factors**, ***n*** **(%)**			
Previous ischemic stroke	23 (22.1)	13 (27.1)	0.50
Arterial hypertension	92 (88.5)	44 (91.7)	0.55
Diabetes mellitus type II	44 (42.3)	23 (47.9)	0.52
Hyperlipidemia	78 (75)	39 (81.3)	0.40
Atrial fibrillation	29 (27.9)	15 (31.3)	0.67
Smoking	17 (16.3)	5 (12.5)	0.54
**Pre-medication**, ***n*** **(%)**			
Antiplatelet therapy	44 (42.7)	14 (29.2)	0.11
Anticoagulation	3 (2.9)	4 (8.3)	0.21
**Large vessel occlusion**, ***n*** **(%)**			
Any	8 (7.7)	20 (41.7)	<0.0001
Terminal internal carotid artery	3 (2.9)	4 (8.3)	
Middle cerebral artery	4 (3.9)	12 (25)	
Basilar artery	0 (0)	1 (2.1)	
Other	1 (1)	3 (6.3)	
**Process times, median (IQR)**			
Door-to-imaging, min	17 (24)	18 (20)	0.40
Door-to-needle, min	74 (57)	67 (39.5)	0.20
Onset-to-treatment, min	210 (45)	228 (58)	0.02
Door-to-consult, min	18 (23)	–	
Teleconsult duration, min^*^	10 (13)	–	
**Stroke etiology**, ***n*** **(%)**			
Toast classification			<0.0001
Large-artery atherosclerosis	8 (7.7)	20 (41.7)	
Small-vessel occlusion	9 (8.7)	4 (8.3)	
Cardioembolism	20 (19.2)	17 (35.4)	
Other determined etiology	1 (1)	1 (2.1)	
Undetermined etiology	66 (63.5)	2 (4.2)	
Stroke mimics	3 (2.9)	-	0.55

*σ, standard deviation; $\bar{x}$, mean; IQR, interquartile range; NIHSS, National Institutes of Health Stroke Scale; mRS, modified Rankin Scale*.

* *According to data from 42 telestroke patients*.

### Intracerebral Hemorrhage

There were no differences concerning sICH following IVT between patients who primarily presented to the spoke sites and those who presented to the hub site [5/104 (4.9) vs. 3/48 (6.3%); *p* = 0.71]. Neither there was a difference in the radiographic evidence of any ICH [9/104 (8.7%) vs. 8/48 (16.7%); *p* = 0.15]. Data on follow-up CT scans were missing in one patient who was treated at the spoke site and who died before follow-up CT. Applying the worst-case scenario, there was still no difference in terms of sICH between both groups [6/104 (5.8) vs. 3/48 (6.3%); *p* = 1.0].

Three of 48 (6.25%) patients treated at the hub site experienced subarachnoid hemorrhage, one of which was considered symptomatic and occurred in addition to ICH. When we considered these bleeding complications in the bivariate analysis, there were fewer intracranial hemorrhages following IVT in patients treated at the spoke sites than in patients treated at the hub site [9/104 (8.7) vs. 10/48 (20.8%); *p* = 0.037].

The multivariable model did not reveal an association between telestroke consultation and sICH following IVT (*p* = 0.5). The results remained the same when we considered only variables that were unbalanced among the study groups (*p* = 0.84). Only atrial fibrillation was associated with sICH (OR: 20.57, 95% CI: 2.38–178.1; *p* = 0.006).

### Short-Term Efficacy Outcomes

Functional outcome data was missing in two patients treated at the spoke sites. No differences were evident in terms of favorable functional outcome at discharge between patients receiving IVT at the spoke sites and the hub site [45/102 (44.1) vs. 19/48 (39.6%); *p* = 0.6].

After adjusting for known covariates in the logistic regression model, age (OR: 0.93, 95% CI: 0.89–0.97; *p* = 0.001), baseline NIHSS (OR: 0.76, 95% CI: 0.67–0.86; *p* < 0.001) and baseline ASPECTS (OR: 1.93, 95% CI: 1.04–3.57; *p* = 0.036) emerged as predictors of favorable functional outcome at discharge, but not telestroke consultation (*p* = 0.68). The results remained the same when we kept all covariates in the model.

Patients treated at the spoke sites appeared to be more frequently discharged to home and less frequently discharged to acute rehabilitation than patients treated at stroke center [43/102 (42.2) vs. 15/48 (31.3%) and 31/102 (30.4) vs. 23/48 (47.9%), respectively]; however, this trend did not reach statistical significance.

At discharge, 10/104 (9.6%) patients in the telestroke group and 4/48 (8.3%) patients in the stroke center group were deceased with no differences in bivariate analysis (*p* = 1.0). Table 2 provides a summary of patient outcomes.

**Table 2:** Safety and short-term efficacy parameters.

	**Telestroke (*n* = 104)**	**Stroke center (*n* = 48)**	***p-*value**
**Safety**, ***n*** **(%)**			
Symptomatic intracerebral hemorrhage^a^	5 (4.9)	3 (6.3)	0.71
Any intracerebral hemorrhage^a^	9 (8.7)	8 (16.7)	0.15
HI1	2 (1.9)	1 (2.1)	
HI2	5 (4.8)	1 (2.1)	
PH1	1 (1.0)	2 (4.2)	
PH2	1 (1.0)	4 (8.3)	
Any intracranial hemorrhage^a^	9 (8.7)	10 (20.8)	0.04
**Short-term efficacy**			
NIHSS at discharge, median (IQR)	3 (5.5)	2.5 (5.75)	0.92
mRS at discharge, median (IQR)^b^	3 (3)	3 (2.75)	0.92
mRS 0-2, *n* (%)	45 (44.1)	19 (39.6)	0.6
Discharge disposition, *n* (%)^b^			0.28
Home	43 (42.2)	15 (31.3)	
Acute rehabilitation	31 (30.4)	23 (47.9)	
Nursing facility	15 (14.7)	4 (8.3)	
Hospital transfer	3 (2.9)	2 (4.2)	
In-hospital mortality, *n* (%)	10 (9.6)	4 (8.3)	1.0

a *One telestroke patient died before follow-up CT*.

b *Missing data on discharge location in two telestroke patients*.

*IQR, interquartile range; NIHSS, National Institutes of Health Stroke Scale; mRS, modified Rankin Scale; HI, hemorrhagic infarction; PH, parenchymal hemorrhage*.

## Discussion

The findings of this observational study suggest that IVT delivered through a hub-and-spoke telestroke network is safe in the expanded 3- to 4.5-h time window. sICH rate of 4.9% at spoke sites was comparatively low in our network and equivalent to the rates of 5.3% reported in the randomized controlled European Cooperative Acute Stroke Study (ECASS) III study and of 3.9% in the SITS-UTMOST and 4.5% in the SITS-ISTR registries (12, 16, 17). Moreover, any intracerebral bleeding occurred less frequently in patients with telestroke and was quite low (i.e., 8.7%) when compared with the incidence rate reported in the population of the ECASS III study (i.e., 27.0%).

The median door-to-needle time at the telestroke spoke sites (i.e., 74 min) was slightly longer than that achieved at the hub site. Longer in-hospital treatment times have also been observed in general patients with stroke who were treated in the expanded 3- to 4.5-h window compared with the 3-h window (16, 17). Potential loss of time at the spoke sites could yet be still attributed to the teleconsultation itself including video-consult initiation and completion. However, with a median of 10 min, teleconsult time was shorter than reported in other large telestroke networks ranging between 14 and 35 min (18–20). Moreover, disregarding the teleconsult duration, door-to-needle times at spoke sites appeared to be comparable to that at the hub site suggesting that in-hospital operational processes of patients eligible to IVT can be established at telestroke units, just as it is for in-person treatment at dedicated stroke centers. Most patients with telestroke in our study also met the proposed door-to-imaging goal of 25 min or less for suspected patients with stroke (21).

As recommended by current AHA guidelines, continuous quality improvement activities are expected to facilitate quality, performance, and outcomes of stroke care provided at telestroke sites (4). In our hub-and-spoke telestroke network, data on stroke quality measures are continuously collected and analyzed and stroke-specific care procedures such as adequate diagnostic and medical treatment, dysphagia screening, and early implementation of rehabilitation are audited regularly by in-person visits at the spoke sites. Lastly, data reported in this observational study originates from the years 2014 and 2015 and increasing on-site stroke experience at spoke sites may have further led to improvement in telestroke process metrics as we were able to show in a recent publication (5). Door-to-needle times improved to an average of 52 min that complies with the 60-min target recommended by current stroke guidelines (13, 14).

Comparability of functional outcome and thus efficacy of IVT is limited by the mRS availability in our telestroke network. Patient outcomes were regularly measured using mRS at discharge and in-hospital mortality, which have been still recommended as short-term proxies for the functional outcome (4). By that, a favorable functional outcome was observed in almost every second patient with telestroke exposed to IVT that was comparable to the corresponding rate in patients directly treated at the hub site. Moreover, given the fact that the ECASS III study and the SITS-ISTR and SITS-UTMOST registries obtained modified Rankin scores at 3 months (mRS ≤ 2: 66.5, 65, and 62.7%, respectively), the frequency of favorable functional outcome seen in our telestroke cohort appears realistic (12, 16, 17). The same applies for in-hospital mortality in our study that was between that in ECASS III (i.e., 6.7%), SITS-ISTR (i.e., 11.1%), and in SITS-UTMOST (i.e., 12%).

Large vessel occlusion was detected more frequently in patients directly admitted to the stroke center than in patients with telestroke (41.7 vs. 7.7%). Considering similar baseline stroke severity in both groups, this difference might be rather related to the infrequent performance of CT angiography at spoke sites during the study period. Acute vessel imaging in patients with acute stroke potentially eligible for reperfusion therapies was not implemented as standard of care in our telestroke network until the first efficacy data for EVT were presented in 2015 (1). We, therefore, do not assume that this imbalance in vessel occlusion status has confounded our findings on ICH or functional outcomes, which is also supported by the results of our multivariable model. In the meantime, routine implementation of CT angiography in the acute stroke workup has led to equivalent large vessel occlusion detection rates and allows proper identification of those potentially amenable to EVT (5).

Our study has limitations that largely arise from its observational design. However, aside from slightly longer onset-to-treatment times in patients directly admitted to the hub center, there was homogeneity in terms of demographics, vascular risk factors, baseline stroke severity and initial radiographic extend of early ischemic changes providing a sufficient degree of comparability across both cohorts. Moreover, a randomized controlled trial of IVT in the expanded time window in the telestroke setting would potentially compromise treatment times and there is still the notion that IVT should be initiated at the nearest hospital equipped with tPA (13). There was a substantial amount of missing data regarding the duration of the teleconsultation; however, we do not expect that this has influenced outcomes chosen in this study. Also, our findings are not generalizable to telestroke networks other than hub-and-spoke models.

In conclusion, our observational data supports the equivalence of safety and short-term efficacy of IVT in the expanded 3- to 4.5-h times window between telestroke units and a dedicated stroke center. Considering recent data on further expansion of the treatment window, there is a need to explore the delivery of IVT through telestroke networks using advanced imaging modalities (10, 11).

## Data Availability Statement

The raw data supporting the conclusions of this article will be made available by the authors, without undue reservation.

## Ethics Statement

The studies involving human participants were reviewed and approved by Ethics Committee (EK) of the Technische Universitaet Dresden. Written informed consent for participation was not required for this study in accordance with the national legislation and the institutional requirements.

## Author Contributions

KB and JB: conceptualization and supervision. MF, KB, and JB: methodology. MF and KB: statistical analysis. CW, HR, VP, KB, and JB: resources. ES, MF, AA, SW, CW, KB, and JB: data curation. ES: writing—original draft preparation. ES, L-PP, TS, HR, VP, KB, and JB: writing—review and editing. All authors have read and agreed to the published version of the manuscript.

## Conflict of Interest

The authors declare that the research was conducted in the absence of any commercial or financial relationships that could be construed as a potential conflict of interest.

## Publisher's Note

All claims expressed in this article are solely those of the authors and do not necessarily represent those of their affiliated organizations, or those of the publisher, the editors and the reviewers. Any product that may be evaluated in this article, or claim that may be made by its manufacturer, is not guaranteed or endorsed by the publisher.
